# Superradiant Cherenkov–wakefield radiation as THz source for FEL facilities

**DOI:** 10.1107/S1600577520014058

**Published:** 2021-01-01

**Authors:** Klaus Floettmann, Francois Lemery, Martin Dohlus, Michaela Marx, Vasili Tsakanov, Mikayel Ivanyan

**Affiliations:** a Deutsches Elektronen-Synchrotron, Notkestraße 85, 22607 Hamburg, Germany; b CANDLE SRI, 31 Acharyan Str., 0040 Yerevan, Armenia

**Keywords:** THz source, Cherenkov radiation, dielectric tube, Vlasov antenna

## Abstract

An electron beam passing through a tube of small inner diameter which is lined on the inside with a dielectric layer will radiate energy in the THz range due to the interaction with the boundary. The resonant enhancement of certain frequencies is conditioned by structure parameters such as tube radius and the permittivity and thickness of the dielectric layer. In low-loss structures narrow-band radiation is generated which can be coupled out by suitable antennas. For higher frequencies, the coupling to the resistive outer metal layer becomes increasingly important. The losses in the outer layer prohibit reaching higher frequencies with narrow-band conditions. Instead, short broad-band pulses can be generated with still attractive power levels. In the first section of the paper, a general theory of the impedance of a two-layer structure is presented and the coupling to the outer resistive layer is discussed. Approximate relations for the radiated energy, power and pulse length for a set of structure parameters are derived and compared with numerical results in the following section. Finally, the first numerical result of the out-coupling of the radiation by means of a Vlasov antenna and estimates of the achieved beam quality are presented.

## Abstract 2

The generation of THz radiation in the frequency range of 0.1 THz up to >10 THz by means of Cherenkov–wakefield radiation in dielectric lined tubes is discussed. Achievable power levels and pulse structures fulfill the needs of pump–probe type experiments at modern XFEL facilities. Cherenkov–wakefield radiators hence offer a simple, inexpensive and versatile option for the generation of THz radiation over a wide range of parameters.

## 1. Introduction   

X-ray free-electron lasers (XFELs) are the brightest, tunable sources of short X-ray pulses available for basic scientific research. In order to unfold the full scientific potential of FELs it is, however, mandatory to complement the X-ray sources with suitable pump sources for pump–probe type experiments. The selective (non-)linear excitation of matter by electromagnetic radiation in the sub-THz to THz range (few meV photon energies) enables the deposition of energy into specific low-frequency modes of the material and thus allows to control the impact of various degrees of freedom onto material properties (Dhillon *et al.*, 2017 ▸). The quest for suitable THz sources for pump–probe-type experiments at XFEL facilities is thus a major development goal.

User requirements for the envisaged pump–probe experiments cover a broad parameter range of THz beam properties, for example frequencies spanning from 0.1 THz up to 30 THz combined with pulse energies of 3 mJ at 0.1 THz down to 0.03 µJ at 30 THz (Zalden *et al.*, 2018 ▸). Here it is assumed that a focused beam size with diameter of the wavelength can be achieved at the sample, so that the pulse energy scales quadratically with the frequency and field gradients in excess of 100 MV m^−1^ or equivalent magnetic fields of ∼0.3 T are reached. A nearly diffraction-limited transverse beam quality is hence desirable. Moreover, a suitable THz source has to be synchronized to the XFEL facility with a low temporal jitter (below 20 fs at 5 THz) and it has to be able to deliver THz pulses at the operational repetition rate of the XFEL, so that the full potential of the facility can be employed.

Electron-beam-based THz sources can in principle cope with the high power, repetition rate, and frequency requirements. The freely available spent beam after a SASE undulator is an attractive option for this purpose, because it still has a high quality and a high beam power. Moreover, it is naturally synchronized to the X-ray pulses and can fulfill all repetition rate requirements. [For a detailed discussion on the compensation of path length differences between THz and X-ray pulses see Tanikawa *et al.* (2018 ▸,2019 ▸) and Zhang *et al.* (2019 ▸).]

At the XUV FEL FLASH, for example, a nine-period electromagnetic undulator with 40 cm period length is installed behind the SASE undulator (Borisov *et al.*, 2006 ▸; Morozov *et al.*, 2007 ▸). The generated THz radiation in the range 1.5–30 THz is transported through a ∼65 m-long evacuated beamline to the experimental chamber where it meets the XUV pulse on the sample (Gensch *et al.*, 2008 ▸; Willner, 2008 ▸). Six refocusing mirrors in combination with planar mirrors keep the beam size under control and direct the beam to the experiment. A variable delay line in the XUV path allows for adjusting the relative timing of pump and probe beam.

Following the design ideas of FLASH the installation of a special undulator for the generation of THz radiation behind a SASE undulator is discussed for the European XFEL by Tanikawa *et al.* (2018 ▸, 2019 ▸) and for LCLS-II by Zhang *et al.* (2019 ▸). However, the high beam energy of XFEL facilities (>10 GeV) can require a total undulator length of 10 m with peak fields of up to 7.3 T and a period length of 1 m to comply with only a portion of the requested THz parameters. While such an undulator appears to be technically feasible with state-of-the-art superconducting technology, the cost and complexity of such a device is not attractive.

Another conceivable option is the installation of a separate accelerator near the experimental hutch, because THz radiation can be generated with conventional undulator parameters already at some 10 MeV beam energy. Also the exploitation of a SASE process in the THz range is possible at these low electron beam energies (Schneidmiller *et al.*, 2012 ▸; Vardanyan *et al.*, 2014 ▸). Compact accelerators based on advanced concepts like, for example, plasma acceleration are however not yet able to deal with the beam quality, charge and repetition rate requirements, and cost, size and complexity of the system, based on conventional or advanced accelerator technology, are in any case very significant.

In this paper we consider another option which has not yet been discussed in detail with respect to the broad user requirements of XFEL pump–probe experiments, *i.e.* the utilization of superradiant THz radiation which is created by electron beams passing through vacuum pipes which are coated on the inside with a layer of, for example, a dielectric material (Lemery *et al.*, 2019 ▸). The radiation process is treated in the literature as Cherenkov radiation in media with boundary conditions (for example, Bolotovski, 1961 ▸, 1962 ▸), but also in terms of wakefields (for example, Ng, 1990 ▸), where the focus is stronger on the effect of the boundaries onto the electron beam (induced energy spread and transverse forces). Both radiation and wakefield effects as the induced energy spread are firmly described by theoretical models and by numerical results and have been experimentally demonstrated, primarily in the frequency range 0.1–1 THz (see, for example, Hüning *et al.*, 2002 ▸; Cook *et al.*, 2009 ▸; Antipov *et al.*, 2013 ▸, 2016 ▸; Smirnov *et al.*, 2015 ▸).

At relativistic beam energies the radiation wavelength in these structures becomes independent of the beam energy and is conditioned only by parameters of the vacuum tube, making it possible to use the spent electron beam after a SASE undulator. Especially for the lower frequency range (<10 THz) this technology offers a simple and cost-effective solution. In the following section a generalized representation of the impedance of two-layer vacuum tubes is presented. The bandwidth of the radiation is determined by losses in the dielectric and the coupling to the outer metallic layer which limits the narrow-band characteristics to the lower frequency range. Based on a discussion of the radiation characteristics, relations to estimate radiated energy, power and pulse length for a set of structure parameters are derived and compared with numerical results in the following section. Finally, first numerical result concerning the out-coupling of the radiation by means of a Vlasov antenna and first estimates of the achieved beam quality are presented.

## 2. Impedance of a two-layer tube   

**Figure 1:**
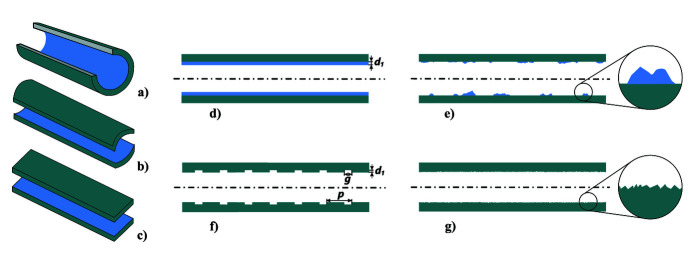
Possible radiator configurations are, for example, round tubes (*a*), and curved (*b*) or flat (*c*) parallel plate waveguides (PPWG). Parallel plate waveguides offer a tuning possibility via the distance of the plates but suffer from a reduced mode confinement as compared with the geometrical simple round tubes. While the outer structure of the radiator is a highly conducting metal, the inner coating can be a thin dielectric layer or a metal with low conductivity (*d*), but also patches of a dielectric material (*e*), regular geometrical structures like corrugations in a metal (*f*) or simply a rough metal surface (*g*). The last three cases are examples of meta-materials, because the radiation characteristics are described by an effective permittivity.

A two-layer tube consists of an outer conducting tube which is coated on the inside by a thin layer of an electromagnetic meta-material. The term meta-material is justified here because the layer can for example be a low-conductivity metal (layer thickness smaller than the skin depth), or a dielectric material or a purely geometrical structure, like regular radial grooves in a metal (corrugated structure), or simply a statistical rough metal surface (Novokhatsky *et al.*, 1998 ▸), see Fig. 1 ▸. All layers are represented by an effective permittivity of the material and an effective layer thickness. For relatively thick, smooth layers the permittivity is in general the permittivity of the bulk material, but for thin layers the permittivity may deviate from the permittivity of the bulk material and depend on details of the surface morphology.

An example are corrugated structures with radial grooves of gap width *g*, depth *d*_1_ and period *p* in a metal base (Novokhaski & Mosnier, 1997 ▸). The radiation characteristics of the structures are described by an effective permittivity 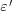, related to the structure parameters by 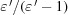 = 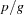, the layer thickness *d*_1_ and the inner radius of the tube *r*_1_. The simple geometry of round tubes (top left) simplifies the theoretical treatment of radiating structures. Besides, round tubes offer a good mode confinement but they are limited in their tuning possibilities. Parallel-plate waveguides (bottom left) on the other hand can be tuned by variation of the plate distance, but the mode confinement is limited. Curved parallel plate waveguides improve the mode confinement and are tunable over a certain parameter range.

We present first a generalized description of the round tube impedance; the pure dielectric layer (dielectric lined) and the metallic layer (bimetallic) case are treated as limiting cases of this general form. For the radiation production low-loss structures are preferable due to the lower bandwidth of the radiation. Small losses in the dielectric layer determine the bandwidth of the radiation in the low-frequency range, while for higher frequencies the coupling to the outer metallic layer becomes relevant as will be discussed below.

A charged particle traveling through a (two-layer) tube interacts with the surrounding material through its space charge field, which is described in the laboratory frame by an infinite spectrum of waves traveling in the radial direction. When a wave hits a material boundary it will be (partially) reflected, transmitted and/or diffracted. If the boundary is metallic the evanescent wave inside the metal will experience resistive losses which lead to a retarding force acting back onto the charged particle. Also in the case of a dielectric boundary a retarding force acts on the charged particle when the Cherenkov condition 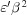 > 1 is fulfilled for the partial wave in the dielectric (Schächter & Schieber, 1997 ▸). Here β is the phase velocity of the wave which corresponds to the velocity of the charged particle.

In a two-layer tube the radiation fields interact also with the second boundary and the reflections on the interfaces lead to the general appearance of narrow-band resonances with typical frequencies in the lower THz range.

The fields near the axis of an arbitrary cylindrical symmetric structure can be expanded in terms of transverse electric (TE) and transverse magnetic (TM), or as hybrid electromagnetic (HEM) mode. Field matching at the boundaries leads to solutions for two-layer (or general multi-layer) structures (Ivanyan *et al.*, 2008 ▸). The following discussion concentrates on the fundamental TM mode at relativistic energies, *i.e.* 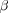 = 1. In the case that the second layer is treated as a perfect metal with infinite conductivity, the longitudinal impedance of an arbitrary nonmagnetic lining can be written as (Ivanyan & Tsakanov, 2004 ▸; Ivanyan *et al.*, 2008 ▸)
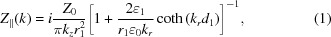
with the vacuum impedance 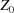 = 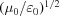, the longitudinal propagation constant 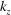 (which matches the free-space propagation constant 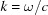), and the transverse propagation constant in the first layer 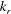 = 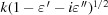 = 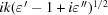. Equation (1) is valid in a high-frequency range when 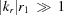.

The effective complex permittivity of the inner layer is described by 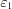 = 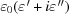, with the vacuum permittivity 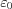. While 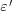 is a measure of the polarizability of the medium, 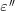 describes losses in the material.

Losses in dielectric layers are small and often ignored; they limit however the shunt impedance and the quality factor of the resonance and are hence included in the derivation below. For a metal the permittivity is defined as 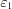 = 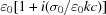, with the static conductivity 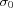, the wavenumber *k* and the speed of light *c*. In contrast to the dielectric layer, losses are dominant in metal layers. Moreover, it is assumed that losses are independent of the frequency in dielectric layers, while they scale inversely with the frequency in metals. Despite these differences, bimetallic and dielectric structures exhibit fundamentally similar resonance characteristics.

Expanding the hyperbolic cotangent term in equation (1) to second order as 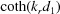 = 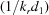 + 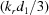 allows to match equation (1) to the general impedance of a parallel resonance circuit (Ivanyan *et al.*, 2014 ▸), 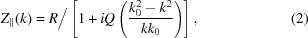
with the resonance wavenumber 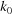 (associated with the resonance frequency 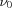), the shunt impedance *R* and the quality factor of the resonator *Q* which is related to the bandwidth of the radiation by 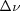 = 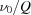. Various asymptotic expressions for the longitudinal monopole impedance of the two-layer dielectric tube are discussed by Ivanyan *et al.* (2020*a* ▸).

**Figure 2:**
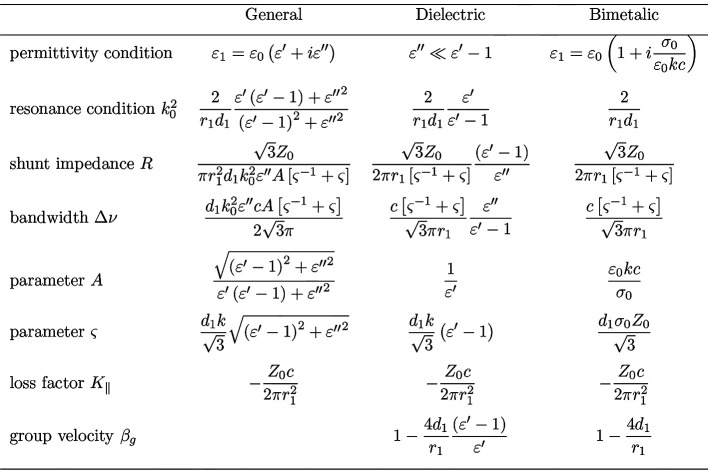
Resonance parameters for the general permittivity case and approximations for the dielectric and the bimetallic case. For details see text.

Figure 2 ▸ summarizes relations resulting from the matching of equations (1) and (2) for the general case and approximations for the dielectric and the bimetallic case (Ivanyan *et al.*, 2014 ▸). The necessary permittivity conditions are listed in the first row. For the resonance condition a thin layer, *i.e.* 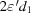 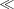 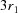, is assumed. For thick layers the relations in the table tend to overestimate the resonance frequency. Parameter *A* combines real and imaginary parts of the permittivity in a way suitable to derive the approximations. Parameter ς relates bandwidth 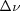 and shunt impedance *R* to the layer thickness 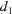 and allows to optimize these parameters, as the bandwidth gets minimal and the shunt impedance maximal for 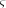 = 1.

The loss factor 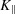 describes the total energy loss per metre of a charged particle traveling through the structure. It is defined as the integral over the complex impedance [equation (2)]
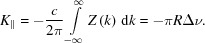
Besides natural constants the loss factor depends only on the inner radius of the structure and is independent of the structure type.

Another important parameter listed in Fig. 2 ▸ is the group velocity (normalized to the speed of light) of the radiation pulse at the resonance frequency, which is not derived from the resonance equation (2) but follows the derivation of Hüning (2002 ▸).

**Figure 3:**
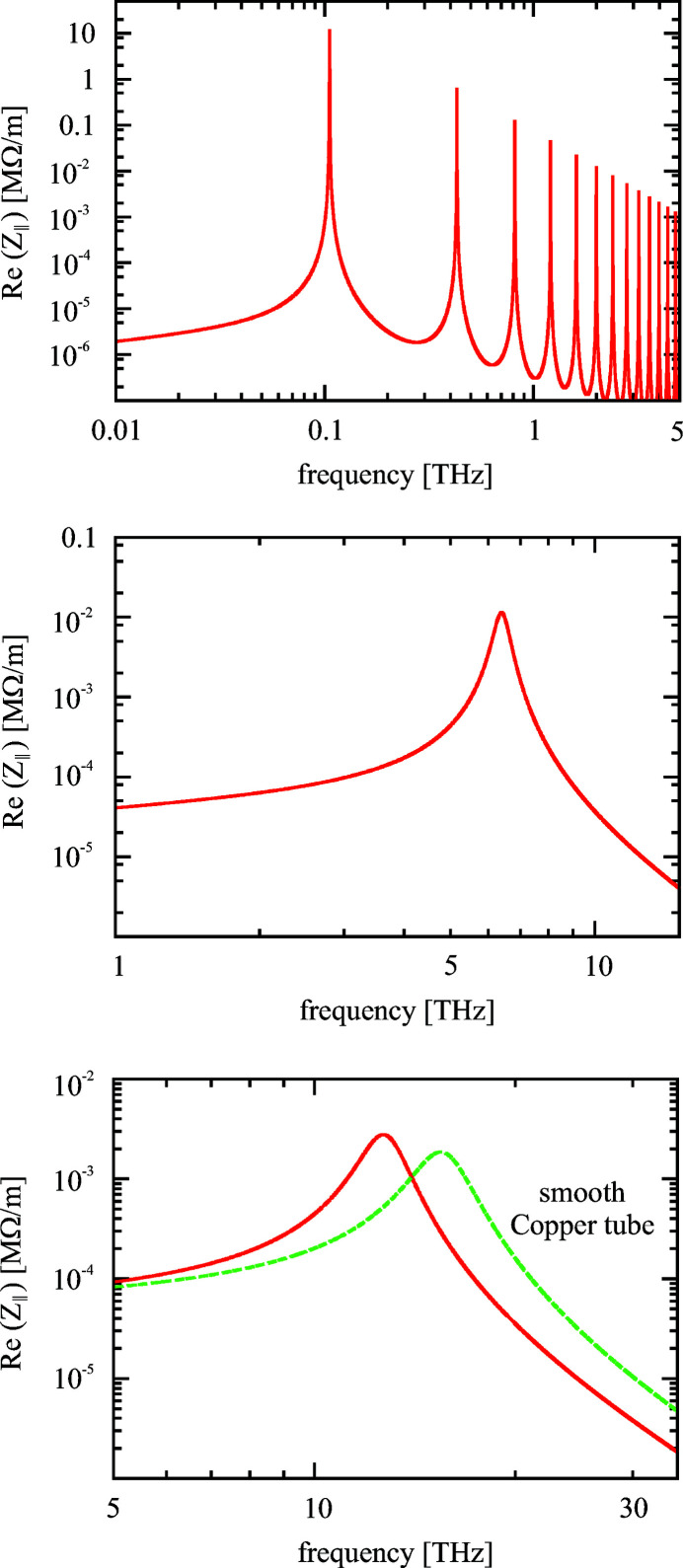
Real part of impedance versus frequency for three different structures in double logarithmic representation. Structure I (top) is matched to 100 GHz. The inner radius 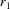 is 2 mm, the permittivity of the dielectric layer 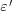 is 3.8 (fused silica), 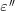 is assumed to be 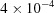 and the layer thickness 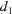 is 225 µm. Structure II (middle) has the following parameters: radius 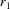 = 1 mm, 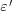 = 1.1, 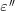 = 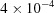 and 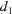 = 1 µm. It reaches a resonance frequency of 6.6 THz. For structure III (bottom) it is finally assumed that the layer thickness 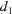 is reduced to 0.1 µm, while all other parameters are identical to the second structure. The resonance frequency of the third structure is 15 THz. The outer layer is in all cases copper with a thickness larger than the skin depth and a conductivity of 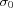 of 58.8 (MΩ m)^−1^. For comparison the impedance of a pure resistive copper tube without surface roughness is included in the bottom plot.

The comparison of structures matched to three different frequencies in Fig. 3 ▸ reveals details beyond the basic analytical description presented above. Table 1 ▸ compiles basic parameters of the structures. At rather thick dielectric layers (top, 100 GHz) many harmonics appear above the fundamental frequency. These resonances are caused by a periodic modulation of the transmission characteristics of the dielectric layer due to the etalon effect, *i.e.* the dielectric layer acts as a Fabry-Perot interferometer.

Mathematically the resonances are related to the periodicity of the hyperbolic cotangent [equation (1)] in the complex plane; they coincide thus with the condition 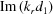 = 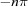, where *n* is an integer. The position of these resonance lines depends hence only on parameters of the dielectric layer and not on the radius of the tube. They are shifted to very high frequencies and low impedance values in the case of thin layers and thus become irrelevant in the thin layer case. Moreover, their dispersion curve levels off and finally does not cross the speed of light anymore. For a detailed discussion on the influence of the layer thickness on the dispersion curve, see Ivanyan *et al.* (2020*b* ▸).

Structures matched to higher frequencies (middle, 6.6 THz; bottom, 15 THz) become increasingly more influenced by the finite conductivity of the outer metal layer. In order to take the resistive losses in the outer layer into account, equation (1) must be extended by a factor Γ (Ivanyan & Tsakanov, 2011 ▸) as
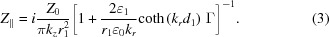
Γ is purely real and equal to 1 for the ideally conducting case, while it can be approximated by 
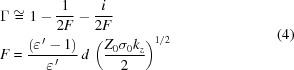
when the resistivity of the outer layer has to be taken into account.

 A resistive metal layer leads to a reduction of the resonance frequency and to an increased bandwidth.

**Figure 4:**
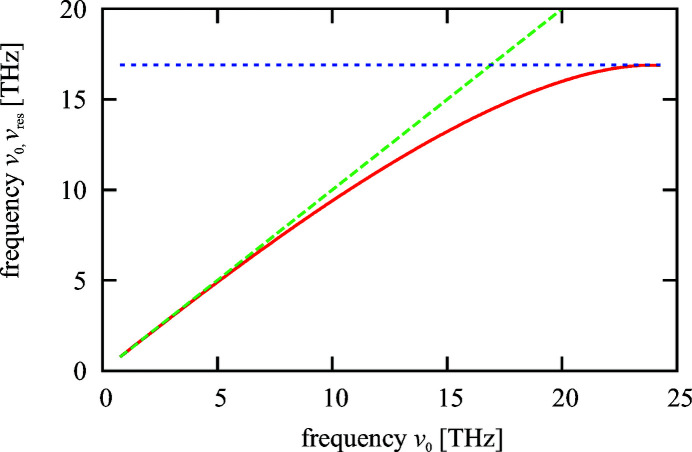
Resonance frequency reduction due to the resistivity of the outer copper layer. The green line represents the expectation according to the resonance condition of Fig. 2 ▸. For the red line the resistivity is taken into account [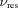 following equation (5)]. The blue line shows the peak position of a pure resistive metal tube (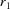 = 1 mm).

The reduction of the resonance frequency is shown in Fig. 4 ▸. The solid red line shows the resonance frequency when the resistivity of the outer copper layer [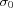 = 58.8 (MΩ m)^−1^] is taken into account in comparison with the expected resonance frequency following the resonance condition of Fig. 2 ▸ (indicated by the broken green line).

The resonance frequency of the coupled system follows the relation 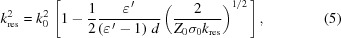
where 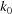 is the resonance wavenumber of the tube with a layer of infinite conductivity (Fig. 2 ▸). Equation (5) can be solved by an iterative procedure. The coupled resonance frequency converges to the peak value of the impedance of a pure metallic tube which is approximately given by
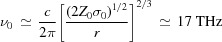
for a copper tube with 1 mm radius. This frequency sets an upper limit for the resonance properties of a two-layer structure for this radius.

The coupling to the resistive layer disturbs the resonance character of the radiation so that the impedance cannot be described by a resonator anymore when the influence of the metal becomes too strong, *i.e.* it develops more and more into a broadband radiation. Note, however, that the loss factor, *i.e.* the total radiated energy, does not change due to the coupling to the resistive layer. The analytical results presented in this section are based on a low-frequency model of the metal permittivity which assumes a frequency-independent conductivity. For higher frequencies (material-dependent >10 THz) the frequency-dependent conductivity following the Drude model of metals should be used. (The Drude model is employed for the impedance plots shown in Fig. 3 ▸.) Within the frequency range discussed here, however, the influence of the Drude model is small. Moreover, the anomalous skin effect may become significant if, for example, the structure shall be cooled in order to increase the conductivity. In that sense the above results are approximate when higher frequencies are considered. More developments are required to improve our understanding of the high-frequency limits.

## 3. Radiation characteristics   

The following discussion concentrates on the dielectric case as presented in Fig. 2 ▸. The focus is on the generation of short pulses, *i.e.* the frequency content of the pulses will be dominated by the 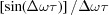 term related to the pulse length τ. The natural bandwidth of the radiation can thus, to first-order, be ignored, and besides the resonance condition only the loss factor and the group velocity are required to estimate radiation parameters.

**Figure 5:**
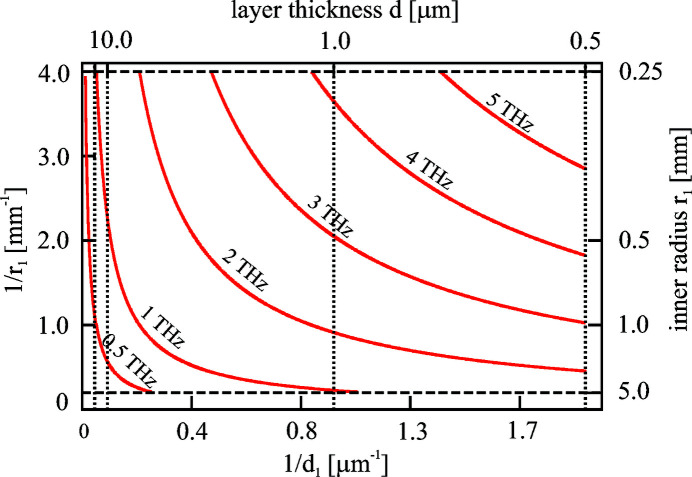
Resonance frequency as a function of inner radius and layer thickness for a dielectric structure with 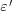 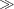 1. For a better separation of the frequency contour lines inverse scales are used.

Figure 5 ▸ shows the resonance frequency for a dielectric structure with large permittivity 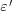 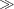 1 as a function of tube radius and layer thickness. In this plot the inner radius ranges from 0.25 to 5 mm, while for the thickness of the inner coating a minimum value of 0.5 µm is chosen.

The lower-frequency range from a few hundred GHz up to a few THz can be easily covered by dielectric structures; it is, however, unclear what the highest achievable frequency for this technology is. Note that the frequencies scale with the ratio 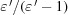. Thus for 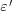 = 2 the 5 THz line would scale up to 10 THz, while it would require 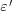 = 1.2 to push it up to 30 THz. Corrugated structures as sketched in Fig. 1 ▸ reach permittivities below 2 and appear hence to be suitable structures for low and medium frequencies. As discussed in the previous section, the finite conductivity of the outer layer (or the base material in case of a corrugated structure) prohibits reaching high frequencies with narrow-band conditions in two-layer structures. Options such as cooling of the metallic tubes to increase the conductivity or more complex structures (multi-layer, photonic band gap) may open a way to higher frequencies but have not yet been investigated in detail. Also, filtering of the broadband radiation of metal tubes is an option as the required power levels decrease with increasing frequency.

Relevant parameters for the high-frequency operation, besides the permittivity, are the layer thickness and the radius of the structures.

**Figure 6:**
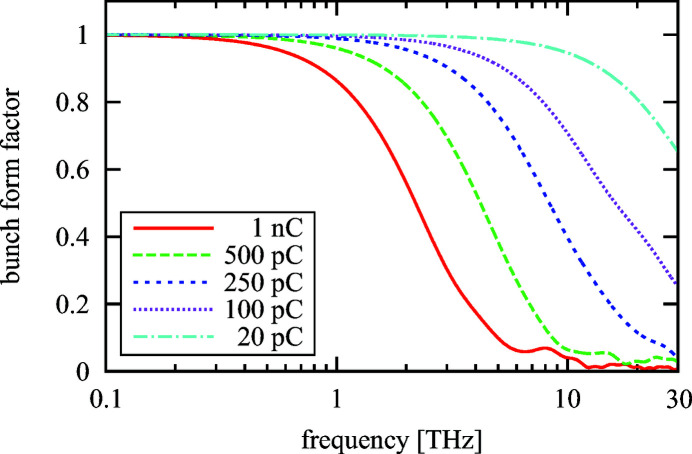
Bunch form factor for different bunch charges at the European XFEL. The particle distributions are generated by start to end simulations up to an energy of 17.5 GeV at the entrance of the undulators. The FEL interaction is thus not yet taken into account (Zagorodnov, 2013 ▸).

For the case of negligible losses, *i.e.* an ideally conducting outer layer and 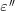 = 0 for the inner layer, the impedance reduces to a delta function and the wakefield is purely monochromatic with an amplitude given by the loss factor as 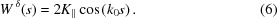
Equation (6) describes the field induced by a single electron as function of the longitudinal position *s* behind and relative to the position of the electron. The field induced by a bunch is proportional to the bunch charge but also to the frequency content of the charge distribution,
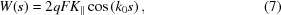
where 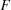 = 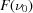 stands for the bunch form factor, *i.e.* the normalized Fourier component at the resonance frequency of the structure. The total energy radiated in a structure of length *L* follows thus as
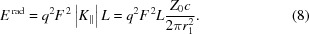
The pulse length of the radiation pulse is related to the group velocity (Fig. 2 ▸) by the relation 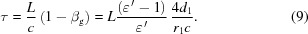
From the resonance condition follows
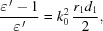
which leads to 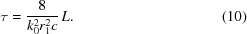
Finally, the power can be calculated as the ratio of the radiated energy equation (8) and pulse length equation (10) as 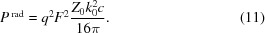
To estimate the tube length required to cope with the user requests as stated by Zalden *et al.* (2018 ▸) the bunch form factor is needed. This is shown in Fig. 6 ▸ for the European XFEL operated at different bunch charges. In the case of operation at lower bunch charge the beam needs to be more strongly compressed to increase the bunch current to the level required for the FEL process and thus a high bunch form factor is reached at higher frequencies. Below ∼1 THz the bunch form factor is close to 1 even for a bunch charge of 1 nC.

Based on this it would, for example, require a structure length of only 66 cm when the structure radius is 2 mm and the bunch charge is 1 nC to generate an energy of 3 mJ [equation (8)] at 0.1 THz (Zalden *et al.*, 2018 ▸). The temporal length of the radiation pulse would be 1 ns (100 cycles) [equation (10)]. The power reached in this case is 3 MW [equation (11)].

The resonance frequency does not enter equation (8); a structure with the same dimensions but different dielectric layer would thus generate the same energy up to a resonant frequency of about 1 THz, when the bunch form factor starts to decrease for the 1 nC case. The pulses at higher frequencies are, however, shorter and the power higher as 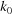 enters into equations (10) and (11). Since less energy is requested at higher frequencies (Zalden *et al.*, 2018 ▸), shorter structures or less charge would still be sufficient to cope with the user requirements.

Note that equations (8) and (10) scale as 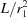; a larger radius thus requires a longer structure to generate the same amount of energy, but the pulse length and the power do not change if the length of the structure is adjusted accordingly.

At higher frequencies the bunch form factor shrinks considerably. Lower bunch charges achieve higher form factors so that it can become advantageous to reduce the bunch charge when radiation above 3 THz shall be generated. On the other hand, a higher bunch charge of 1 nC is preferable for the lower-frequency range but it is not mandatory as longer structures and/or smaller radii are not excluded.

The reduced energy requirements still allow generating sufficient amounts of energy also at higher frequencies. At 6.6 THz (Fig. 3 ▸, middle) only 0.7 µJ of radiation energy are requested which requires less than a centimetre structure length (1 mm radius) at charges between 1 nC and 100 pC.

In view of the discussion of the influence of the finite conductivity of the metallic layer it is clear that the approximate relations [equations (8)–(11)] are not applicable at higher frequencies. It is nevertheless interesting to note that only short structures (less than a centimetre) would be required to fulfill the user requests. This allows considering very small radii of the structures.

**Figure 7:**
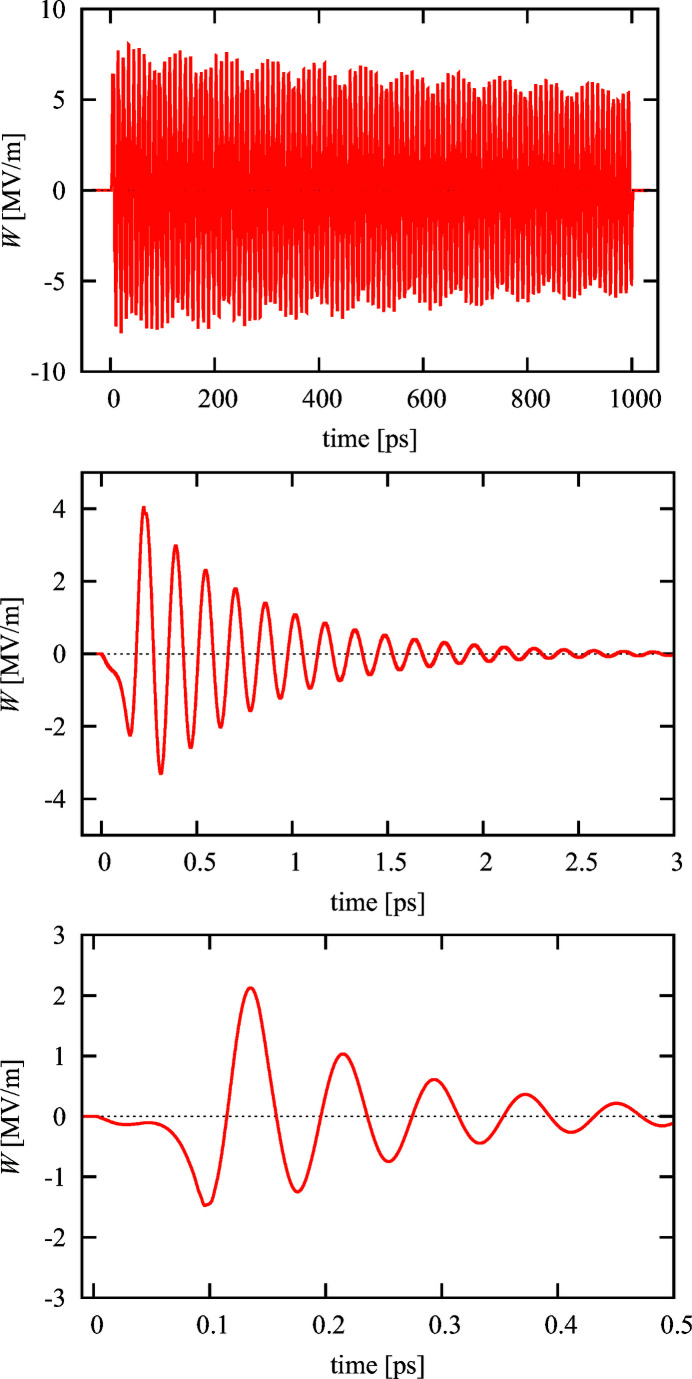
THz wave forms generated by electron beams in dielectric tubes. The parameters of the tubes correspond to the impedance plots shown in Fig. 3 ▸. For the top plot (0.1 THz) a bunch charge of 1 nC is assumed. The pulse amplitude is modulated by the higher harmonics of the structure which are included in this numerical result. The pulse length of 1000 ps corresponds to 66 cm structure length. At 6.6 THz (middle) the pulse is strongly damped and decays with an exponential decay time of about 0.6 ps. Here a bunch charge of 0.5 nC is assumed. The bottom plot shows the 15 THz case with a charge of 0.25 nC. The pulse decays with about 150 fs decay time.

In Fig. 7 ▸, THz waveforms generated by electron bunches for the impedances introduced in Fig. 3 ▸ are compared. The traces are numerically calculated by an inverse Fourier transform of the complex impedance, 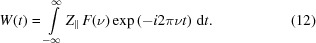
Neither dispersion nor transition effects are included and the time axis corresponds to a wave traveling in vacuum. (The wavelength is reduced inside of the structure due to the lower group velocity.)

At higher frequencies the damping is quite strong, so that a steady state is reached in very short structures. At low frequencies the damping can be very small, so that long nearly sinusoidal waves can be generated. Of course it is also possible to increase the damping for the lower-frequency range if a larger bandwidth is desired.

The considerations above aim to serve as a guideline only. A full design requires numerical simulations including transition effects at the entrance and the exit of the structure. An overhead to account for transport losses has to be included and possible modifications of the pulse structure due to the transport line need to be considered. In the following section some aspects of the out-coupling of the THz radiation and some problems concerning the numerical simulation are briefly presented.

## 4. Out-coupling and transverse beam quality   

An important aspect of the THz generation with dielectric tubes is the out-coupling of the radiation from the generating tube into a free space transport system. Experimentally two concepts have been tested. A straight line extraction of the radiation can be realized by means of tapered structures ending in horn antennas (Cook *et al.*, 2009 ▸; Smirnov *et al.*, 2015 ▸). Since the radiation travels on a straight path overlapping the electron beam (and potentially the FEL radiation), the THz radiation needs to be separated from the electron beam, *e.g.* by a mirror with a hole. A disadvantage of this approach is that the mirror distorts the wavefront of the THz pulse due to the hole. Moreover, diffraction radiation is generated by the electron beam passing through the hole which adds to the THz pulse. If the FEL beam is separated from the electron beam before it enters the THz structure it would of course be possible to employ a dipole magnet for the separation of the THz and the electron beam.

**Figure 8:**
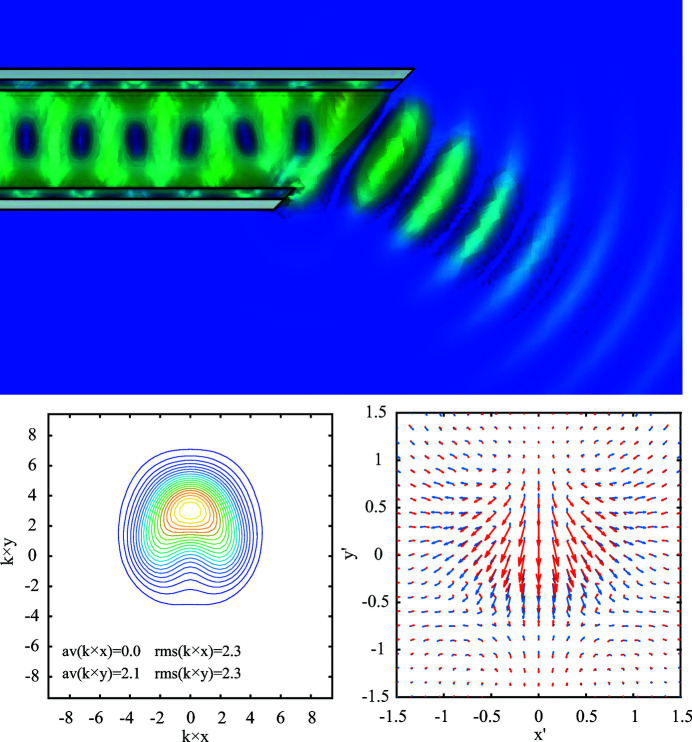
Simulation of a Vlasov antenna with 300 GHz central frequency. The tube has a radius of 450 µm, a layer thickness of 100 µm, and 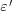 = 3.8 (fused silica). The cut angle has been varied between 25° and 65°. Lower left: perpendicular to the plane component of the Poynting flux through a plane perpendicular to forward direction. The Poynting flux is calculated by a far-field to near-field transformation into the plane with minimal r.m.s. dimensions of the pattern; *k* is the wavenumber. Lower right: vector components of the electric field in the far zone for 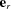 = 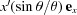 + 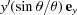 + 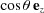 with 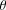 = 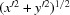. The real and imaginary parts are shown in blue and red.

A second approach is a so-called Vlasov antenna (Vlasov & Orlova, 1974 ▸; Antipov *et al.*, 2016 ▸). Here the tube is cut at an angle which transforms the radiation field into a highly directed beam at an angle relative to the beam axis as shown in Fig. 8 ▸. In the simulations performed with the CST-MWS frequency domain solver for a frequency of 300 GHz a high transmission above 95% of the power was realized at different cut angles (tolerances appear to be uncritical). To characterize the radiation properties the field has been transformed from the finite calculation domain into the far zone, and the coordinate system has been rotated so that the *z*-axis points into the direction of maximal power flux. This corresponds to a counter-clockwise rotation of about 30° around the *y*-axis, that points out of the plane of the diagram, Fig. 8 ▸, top panel.

**Figure 9:**
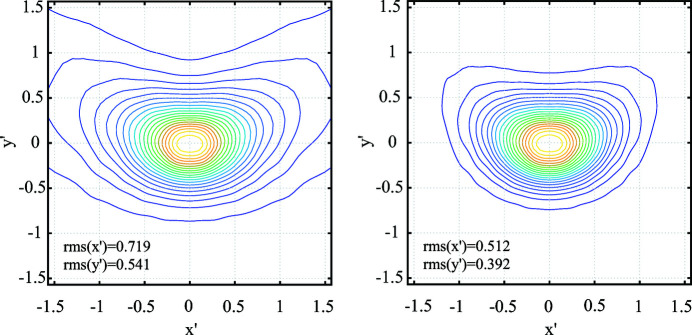
Poynting flux in the far zone. Plotted is the radial component into direction 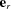 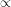 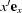 + 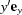 + 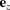 (left) and into direction 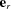 = 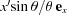 + 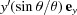 + 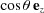 with 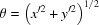 (right).

Figure 9 ▸ presents contour plots of the Poynting flux through a far sphere with different representations of the directional vector 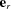 as function of angular coordinates 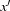 and 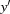. For the left panel of Fig. 9 ▸ these coordinates have the meaning of the slope in the *x*- and *y*-direction as it is convention for beam dynamics. For non-narrow characteristics it is appropriate to use a spherical description as shown in the right panel of Fig. 9 ▸. In this case the spherical angle φ is given by the argument and the angle θ by the absolute value of 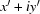. Thus 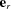 is related to the angles by 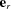 = 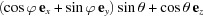 (The difference between both diagrams increases with the distance from the origin.) The polarization of the wave depends on its direction and has even a circular component, see Fig. 8 ▸ (lower right). The Poynting flux in Fig. 8 ▸ (lower left) is obtained by a far- to near-field transformation to a plane *z* = constant, here the plane with minimal r.m.s. dimensions. The beam quality can be estimated from the r.m.s. dimensions in the far (characteristic) and near (waist of the back-propagated beam). It is about twice the diffraction-limited beam quality. Further studies at different frequencies and optimized geometries as well as experimental verifications are required.

## 5. Conclusion and outlook   

We present a generalized treatment of the impedance of a two-layer structure in the form of a matched resonance circuit. Losses in the dielectric layer and especially in the coupled outer metal layer determine frequency content and bandwidth of the radiation. At higher frequencies (very thin dielectric layer, small inner radius) the impedance approaches the typical resistive wakefield impedance of a metal tube. The narrow-band characteristics of the lower frequency range can thus no longer be maintained.

Superradiant Cherenkov–wakefield radiation presents an attractive option as a THz source for FEL facilities especially for the lower-frequency range up to a few THz. The user requirements can be met with moderate geometrical parameters of the tubes. At higher frequencies less energy is requested, so that shorter tubes can be employed which allows to reduce the radius further. In addition, very thin dielectric layers are required to push this technology to higher frequencies.

While robust structures with relatively thick dielectric layers can be made for example from fused quartz tubes which are coated with copper on the outside, the production of thin layers can be based, for example, on sputtering or simple oxidation of a metal tube on the inside. Corrugated structures offer another way to reach a small effective permittivity and a thin layer thickness.

The structures, especially the all-metal structures, are in general robust and will withstand some particle loss and energy deposition (heat load) which may appear during operation. A first study of a corrugated structure for LCLS II (Bane *et al.*, 2017 ▸) indicates manageable heat load problems for the considered parameters. For the examples in this paper a minimum inner radius of 1 mm has been chosen, while considerably smaller radii have been utilized in some experiments (Cook *et al.*, 2009 ▸; Antipov *et al.*, 2013 ▸, 2016 ▸). Operational experience with a high power beam, such as for example the beam of the European XFEL, is however missing at this point in time. Also the experimental investigation of high field effects which might limit the performance requires more attention (O’Shea *et al.*, 2019 ▸). Therefore the maximum frequency which can be generated under these conditions by means of Cherenkov–wakefield radiation is presently still open.

Round tube structures are attractive for their radiation characteristics, but their tuning capability is limited. It is, however, conceivable to install a larger number of tubes on a movable stage, so that not only a substantial frequency range can be covered but also tubes for different pulse lengths or bandwidths can be provided. This flexibility is unique to this technique.

Curved parallel plate waveguides are another radiator option with increased tuning capability. These structures can potentially also produce variable chirped pulses for later THz-pulse compression. Also the generation of two or more colors in a single pulse by passing the beam through structures with different radii is conceivable. Cherenkov–wakefield radiators are hence not only simple and inexpensive in comparison with other options for the generation of THz radiation for pump–probe experiments at FEL facilities. They also offer a high flexibility over a broad range of parameters to cope with various user requests.

## Figures and Tables

**Table 1:** Basic parameters of the structures of Figs. 3 ▸ and 7 ▸; the outer layer is in all cases copper with a thickness larger than the skin depth and a conductivity of 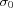 = 58.8 (MΩ m)^−1^

Structure	Radius 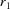 (mm)	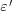	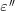	Thickness *d*_1_ (µm)
I	2.0	3.8	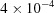	225
II	1.0	1.1	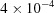	1.0
III	1.0	1.1	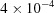	0.1
